# FAVR (Filtering and Annotation of Variants that are Rare): methods to facilitate the analysis of rare germline genetic variants from massively parallel sequencing datasets

**DOI:** 10.1186/1471-2105-14-65

**Published:** 2013-02-25

**Authors:** Bernard J Pope, Tú Nguyen-Dumont, Fabrice Odefrey, Fleur Hammet, Russell Bell, Kayoko Tao, Sean V Tavtigian, David E Goldgar, Andrew Lonie, Melissa C Southey, Daniel J Park

**Affiliations:** 1 Victorian Life Sciences Computation Initiative, The University of Melbourne, 187 Grattan Street Carlton, Melbourne, Victoria 3010, Australia; 2 Genetic Epidemiology Laboratory, Department of Pathology, Medical Building, The University of Melbourne, Melbourne, Victoria 3010, Australia; 3 Huntsman Cancer Institute and Department of Oncological Sciences, University of Utah School of Medicine, Salt Lake City 84112, USA; 4 Department of Dermatology, University of Utah School of Medicine, Salt Lake City 8411, USA

**Keywords:** Massively parallel sequencing, Rare genetic variants, Filtering, Annotation, FAVR

## Abstract

**Background:**

Characterising genetic diversity through the analysis of massively parallel sequencing (MPS) data offers enormous potential to significantly improve our understanding of the genetic basis for observed phenotypes, including predisposition to and progression of complex human disease. Great challenges remain in resolving genetic variants that are genuine from the millions of artefactual signals.

**Results:**

FAVR is a suite of new methods designed to work with commonly used MPS analysis pipelines to assist in the resolution of some of the issues related to the analysis of the vast amount of resulting data, with a focus on relatively rare genetic variants. To the best of our knowledge, no equivalent method has previously been described. The most important and novel aspect of FAVR is the use of signatures in comparator sequence alignment files during variant filtering, and annotation of variants potentially shared between individuals. The FAVR methods use these signatures to facilitate filtering of (i) platform and/or mapping-specific artefacts, (ii) common genetic variants, and, where relevant, (iii) artefacts derived from imbalanced paired-end sequencing, as well as annotation of genetic variants based on evidence of co-occurrence in individuals. We applied conventional variant calling applied to whole-exome sequencing datasets, produced using both SOLiD and TruSeq chemistries, with or without downstream processing by FAVR methods. We demonstrate a 3-fold smaller rare single nucleotide variant shortlist with no detected reduction in sensitivity. This analysis included Sanger sequencing of rare variant signals not evident in dbSNP131, assessment of known variant signal preservation, and comparison of observed and expected rare variant numbers across a range of first cousin pairs. The principles described herein were applied in our recent publication identifying *XRCC2* as a new breast cancer risk gene and have been made publically available as a suite of software tools.

**Conclusions:**

FAVR is a platform-agnostic suite of methods that significantly enhances the analysis of large volumes of sequencing data for the study of rare genetic variants and their influence on phenotypes.

## Background

We applied FAVR principles, described herein, to identify that rare mutations in *XRCC2* increase the risk of human breast cancer, which is to our knowledge the first published report of massively parallel sequencing (MPS) being successfully applied to identify a new complex human disease gene [1].

Rare genetic variants have been proven to contribute a significant fraction of the heritable component of complex human disease [2,3], as well as a range of Mendelian disorders [4].

We can now generate large sequencing datasets cross-referenced to detailed human disease phenotypes [5]. However, such datasets contain millions of disease-unrelated variants and artefacts. Conventional genetic variant calling software e.g. GATK (http://www.broadinstitute.org/gsa/wiki/index.php/The_Genome_Analysis_Toolkit) use Bayesian co-variate analysis methods to determine the probability that variants are real based on metrics such as mapping quality score, base quality metrics, read depth, variant read frequency, mate-pairing, bi-directionality, and co-occurrence of nearby deviations from the reference genomic sequence. These methods can apply optimally determined metrics to a given test specimen but do not use individual variant comparisons across multiple specimens. At the outset of our whole-exome sequencing work, we were concerned that the majority of variants ‘called’ using GATK appeared suspicious when viewed alongside other whole-exome data tracks derived from identical chemistry and bioinformatic processing pipelines (Additional file 1: Figure S1). Many of these appeared to be quite common across our samples and yet did not feature in HapMap project databases in which Sanger sequencing-based determination of variants had been applied. Frequency of such signals across specimens and the proportion of aligned reads exhibiting signal for a given specimen varied widely. Of concern, it was not readily apparent how the co-variate analyses performed by conventional variant calling software could be used to filter out such artefacts without seriously compromising the sensitivity of variant calling. The potential filtering metrics of these artefacts were often in the ranges observed for proven variants.

Further, we and others have observed that a large number of artefactual variants derive from shorter reads following SOLiD4 paired-end sequencing (which yields 50 bases and 35 bases reads at either end of library fragments) [6].

Herein, we describe and validate FAVR methods for the shortlisting of rare, germline variants of potential disease-relevance, with dramatically improved specificity and without compromising sensitivity, based on observations of signals from sequence alignment files that are detectable by comparison across but not necessarily within datasets.

## Methods

### Subjects

The subjects were part of a collaborative multiple-case, early-onset, breast cancer family exome sequencing project and were selected from the international Breast Cancer Family Registry. The subjects selected in the present study were pairs of affected first cousins, from ten families.

### Whole-exome sequencing

Families 1 to 5 were sequenced on a SOLiD4 instrument (Life Technologies) and families 6 to 10 were sequenced on a HiSeq instrument (Illumina).

Libraries were prepared for paired-end seqeuncing following SOLiD ((Life Technologies) and TruSeq (Illumina) protocols respectively. Exome-capture was performed with the Nimblegen SeqCap EZv2 (Roche Nimblegen, Inc.) exome-capture DNA kit, then sequenced using the SOLiD4 instrument or the HiSeq instrument.

The sequence data from this study are available via dbGaP (http://www.ncbi.nlm.nih.gov/projects/gap/cgi-bin/study.cgi?study_id=phs000601.v1.p1).

### Pre-FAVR bioinformatic processing

Reads generated using the SOLiD4 were mapped to a human reference (hg19) using Bioscope v1.3.1, locally realigned using GATK v1.0.5336 and duplicates were removed using Picard v1.29 (http://picard.sourceforge.net/index.shtml).

Reads generated on the HiSeq were mapped to hg19 using Novoalign (Novocraft). GATK v1.6 was used for local realignment, duplicate removal and base quality recalibration (GATK TableRecalibration).

Based on the GATK exome analysis pipeline, single nucleotide variants (SNVs) were called using the GATK v1.5 UnifiedGenotyper (with -stand_call_conf=30 -stand_emit_conf=30 -dcov =700 -mbq =17). Variant quality score recalibration was performed using the VariantRecalibrator and ApplyRecalibration tools (default parameters). Raw variant calls were filtered to remove dbSNP131-registered variants (http://www.ncbi.nlm.nih.gov/projects/SNP/) and non-exome variants using BEDtools (http://code.google.com/p/bedtools/). These methods generated alignment files in BAM format [7] and variant lists in VCF format [8]. Variant lists were annotated using ANNOVAR [9].

Additional comparative FAVR analyses using either dbSNP131 or dbSNP135-registered variants were performed after variant calling using GATK v 1.0.5336 (−strand_emit_conf=15 –mbq=15 –mmq=20 –mm40=2 –dcov=700), and are illustrated in Additional file 2: Figure S2.

### FAVR bioinformatic processing

**Figure 1:**
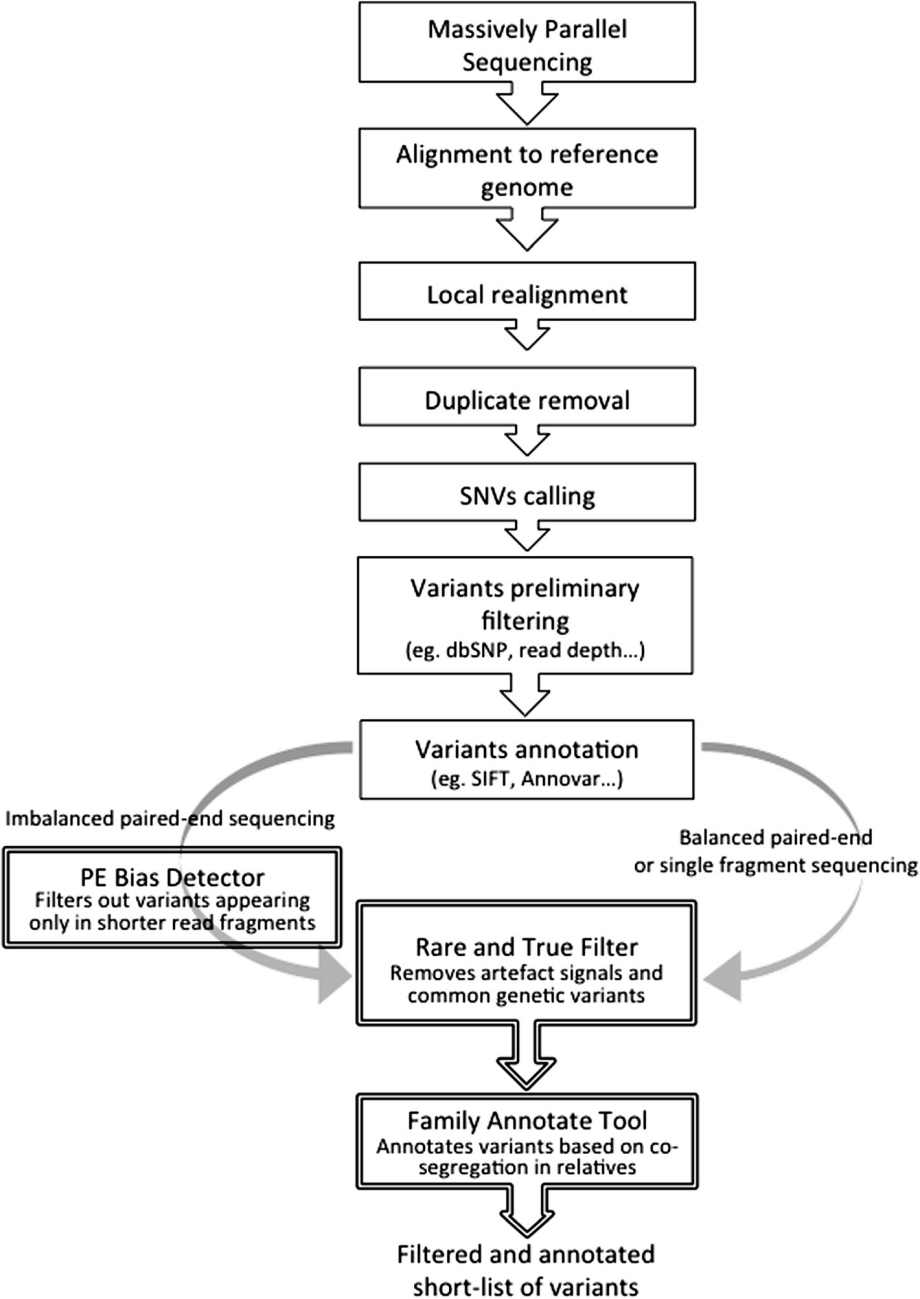
**Standard bioinformatics pipeline for the analysis of rare single nucleotide variants (SNVs).** FAVR methods can be applied to assist the shortlisting of SNVs by using the PE Bias Detector tool, the Rare and True Filter and the Family Annotate Tool. FAVR integrates seamlessly into standard pipelines as it uses widely used file formats (BAM and vcf format files).

FAVR methods were developed as a software suite, including Rare and True Filter, PE Bias Detector, and Family Annotate Tool, and made freely available (https://github.com/bjpop/favr). These methods were applied following pre-FAVR bioinformatic processing and are detailed below (Figure 1). FAVR software suite has been tested on Linux and Mac operating systems.

### FAVR filtering for authentic rare genetic variants

The application of Rare and True Filter makes novel use of signature sequences in BAM files to infer evidence of sequencing/mapping artefacts. Variants observed below a particular frequency in comparator BAM files are kept and all other variants are discarded. Under a rare variant-disease model, the tool does not need to differentiate between mapping/sequencing artefacts and relatively common genetic variants and both are filtered out. The determination of variant frequency is configurable by two command-line parameters. The first parameter is a threshold for the number of reads in a sample that have the same base as the variant in the same position. Samples with a number of variant reads greater than or equal to this threshold are considered variant-like. Having filtered out replicate reads, e.g. using Picard, and using sequencing chemistry with accuracy similar to those of the SOLiD or TruSeq, this threshold can be set to very low levels to remove variants derived from mapping artefacts without sequencing chemistry errors leading to unwanted removal of variants. The second parameter specifies a threshold for the percentage of comparator BAM files (excluding relatives and derived from identical sequencing chemistry and bioinformatic processing) exhibiting evidence of the same variant as the test sample. In this study, we set the first parameter to 1 and set the second parameter to 30%, using 8 comparator BAM files. These are tailorable for application to different platforms or to apply different filtering stringencies.

### FAVR imbalanced pair artefact filtering

Using PE Bias Detector, we can filter out those variants that have been called but which are evident only in short reads of imbalanced pairs. This can be tailored for use with other platforms which exhibit imbalanced paired-end data, but is likely not to be useful in the context of balanced platform data. PE Bias Detector was not required for the analysis of balanced paired-end TruSeq data.

### FAVR annotation of evidence for shared variants

Variants that are found in any reads of any samples from related individuals, for instance, can be annotated using Family Annotate Tool. Depending on the trait inheritance model, this allows shortlisting of genetic variants of particular interest. In the case of a highly penetrant breast cancer gene mutation, for example, a variant might be expected to be observed in multiple affected family members. Similarly, in cases of de novo mutation disorders or rare Mendelian disorders, comparing shared or non-shared variants across family members can inform the process of shortlisting candidate variants of interest. Since this tool uses BAM files as input and not just VCF files, further opportunity is presented to detect rare variants which may be shared between family members, for instance, which would otherwise be missed as false-negatives due to rigid threshold setting.

### First cousins data analysis to assess FAVR processing specificity

To analyse SOLiD-derived datasets, five pairs of first cousins were assessed for the number of SNVs ‘called’ following pre-FAVR bioinformatic processing and following processing with Rare and True Filter, PE Bias Detector or both. In order to assess the specificity of these conditions, we compared observed versus expected SNVs shared between cousins, assuming variants to be rare and genuine and that first cousins are expected to share, on average, 12.5% of their DNA.

The same analyses were performed on TruSeq datasets, derived from five different families.

### Sanger sequencing assessment of FAVR processing specificity

Forty eight rare SNVs not reported in dbSNP131 identified in a range of breast cancer cases involved in the multiple-case breast cancer family exome sequencing project and ‘called’ following FAVR processing were subjected to BigDye Terminator v3.1-based Sanger sequence analysis (Life Technologies). These SNVs were in genes of plausible biological interest with regard to cancer predisposition.

### Known variant testing to assess FAVR processing sensitivity versus pre-FAVR processing

One hundred exonic SNVs were selected in a sampling approach from the Illumina 610 k SNP chip feature list to represent a range of minor allele frequencies across the genome. These were assessed against 12 whole-exome alignment files in the following manner. The assumption was made that if a given SNV was observed in an alignment file at a frequency of greater than or equal to 9%, that specimen was judged to be a ‘true carrier’ of the variant. ‘True carriers’ were excluded from the following analysis. If the variant was apparent in any read in greater than or equal to 30% of the remaining alignment files, the variant would be judged to be an artifact, which would result in a false-negative variant call. We used the filtering thresholds detailed above to gauge the false negative rate of FAVR processing using pre-FAVR filtering as a reference. The vast majority of rare SNVs annotated in current databases have not been validated by Sanger sequencing or by other means to instil confidence and are therefore not appropriate for our purposes here.

## Results and discussion

The principles behind the FAVR methods that are presented in this article were used as part of a body of work which resulted in our recent publication of the first new breast cancer (and to our knowledge, complex human disease) predisposition gene to be identified via MPS analysis, *XRCC2*[1]. In this study, we were presented with a large dataset and needed to yield a manageable shortlist of ‘variants of interest’. As others have done, we removed duplicate reads and performed local re-alignment, filtered for co-ordinates of interest (e.g. the exome), used quality metrics including read depths and frequencies, base and mapping scores, filtered based on prior appearance in databases and minor allele frequencies (e.g. dbSNP), and used in silico predictions of the likely deleterious nature of variants (e.g. SIFT, http://sift.jcvi.org/). Despite these measures, for a majority of variants, we observed evidence of the same signals (signatures) across a range of comparator specimen alignment files, at a range of read depth frequencies and across-sample frequencies. According to a rare variant model, we can remove such ‘variants’ from further consideration either as being too common (yet real) to be of interest or as artefacts (synthetic). We do not differentiate between these classifications, but it is noteworthy that having filtered out features listed in dbSNP131, we have never encountered a homozygous signal for any such ‘variant’ despite their collective apparent high frequencies. This suggests that a majority of such signals are synthetic.

We assume that these signals are the result of mapping/alignment errors in the context of highly similar sequences existing elsewhere in the genome. As to the precise causes, at this point we can only speculate that perhaps polymorphisms and/or sequencing chemistry errors in these regions or in regions of similar sequence could play roles.

Per exome, prior to the development of FAVR methods, we were faced with a shortlist of approximately 1000 SNVs of possible interest. Only approximately one third of these would have been real rare variants, and laborious and expensive Sanger sequencing-based validation would have been required to further shortlist.

FAVR filtering methods compare signals across comparator datasets in a way that has not been described previously. As such, the best performance comparison we felt we could make was based on a conventional bioinformatic analysis pipeline with or without the additional application of FAVR processing (Figure 1). The variant calling software we used, GATK, is among the most widely-used in the field and has been described as representing ‘best practice’ [10]. Testing the sensitivity and specificity of rare SNVs presents certain challenges. Recent iterations of dbSNP, following the incorporation of MPS data, contain a large number of unvalidated rare variants, many of which are likely to be artefacts. As such, simple simulated dataset analyses would not be appropriate for our purpose. Instead, we opted to undertake a series of sampling-based analyses, in which we could be confident of the validity of our reference points, to assess the sensitivity and specificity performances of FAVR methods.

**Figure 2:**
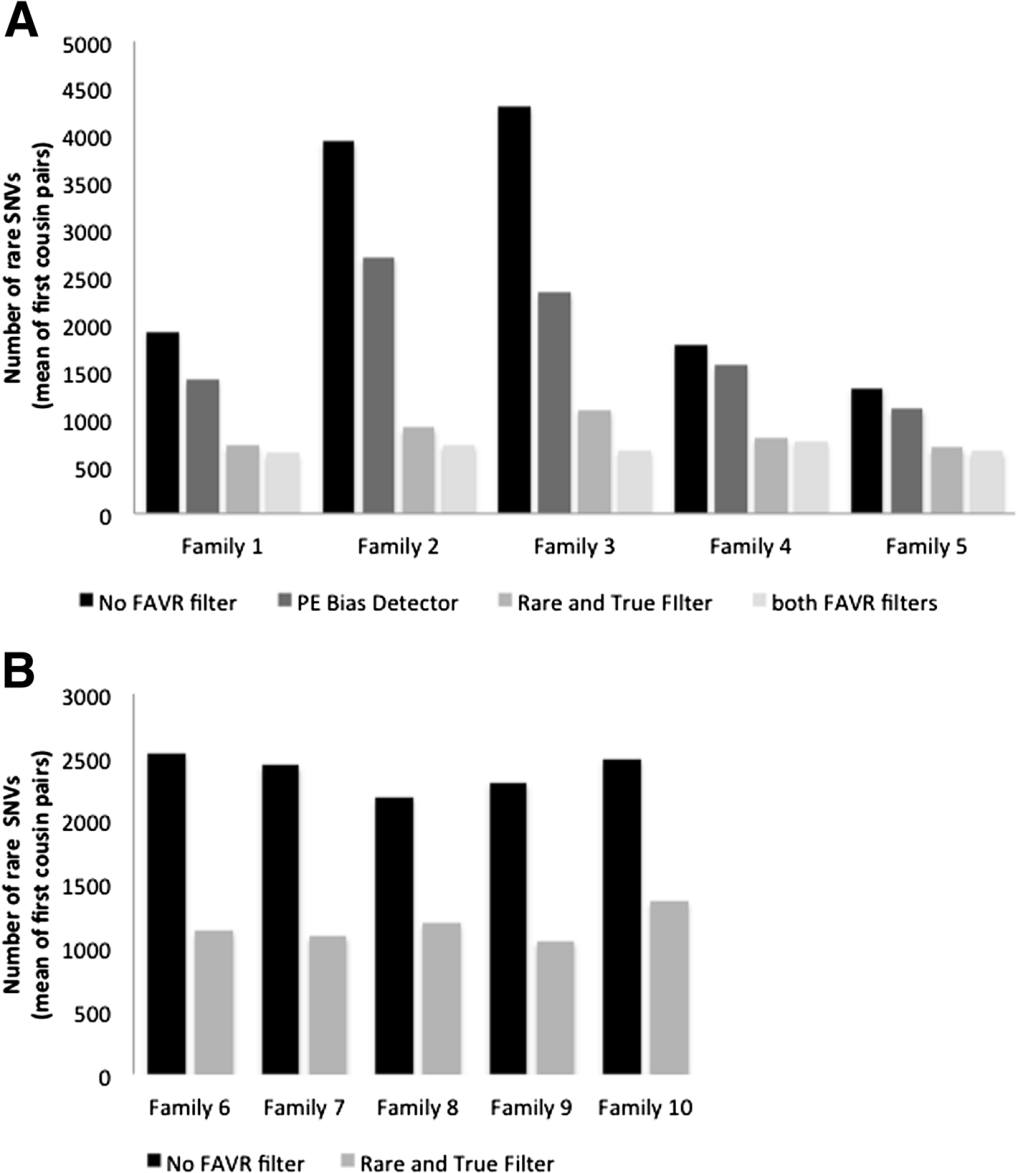
**Number of rare SNVs remaining after different stages of SOLiD and TruSeq sequencing data filtering.** Mean (of first cousin pairs) number of rare SNVs remaining: (**A**) without any further filtering and using the PE Bias Detector Tool only, the Rare and True Filter only, or both tools in five families in which sequencing was performed using the SOLiD chemistry, and (**B**) without any FAVR filtering or using the Rare and True Filter in five families in which sequencing was carried out using the TruSeq chemistry (see Results and discussion). Data were processed according to *Pre-FAVR bioinformatic processing* and further FAVR filtering was applied as described in *FAVR bioinformatic processing* (see Methods).

Our experiments to assess the observed/expected (O/E) shared SNVs between first cousins indicate that the application of FAVR methods dramatically increases variant calling specificity, for sequencing data derived both from the SOLiD and the TruSeq chemistries. The total numbers of variants found in each individual and observed number of shared variants between first cousins pairs at each stage of filtering are reported in Additional file 3: Table S1. Figure 2 illustrates the mean (first cousin pairs) number of SNVs remaining in each of ten families after pre-FAVR processing and following additional application of PE Bias Detector, Rare and True Filter or both. Applied to SOLiD-derived data, the latter three filtering conditions resulted in a mean proportion of variants remaining relative to pre-FAVR processing of 0.74, 0.37 and 0.32, respectively (95% confidence intervals (95% CIs) = [0.62-0.85], [0.26-0.48] and [0.19-0.45]) (Figure 2A). For TruSeq-derived data, the mean proportion of variants remaining after Rare and True Filter was 0.49 (95% CI=[0.44-0.54]) (Figure 2B).

**Figure 3:**
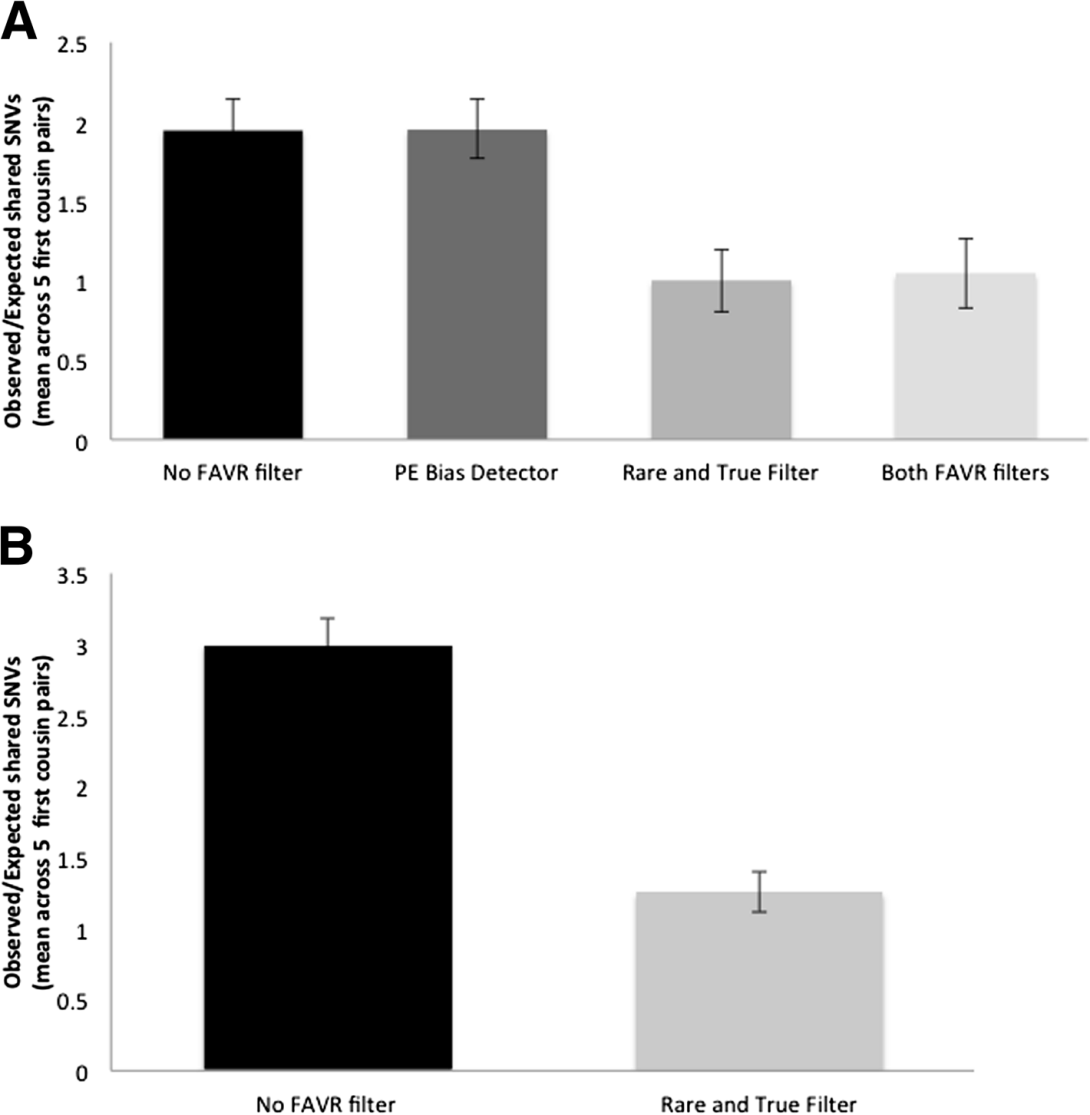
**Observed/Expected (O/E) number of shared SNVs after different stages of SOLiD and TruSeq sequencing data filtering.** Mean (across families) O/E number of shared SNVs, assuming first-cousins share on average 12.5% of their DNA: (**A**) without further filtering, using the PE Bias Detector Tool only, the Rare and True Filter only, or both tools in five families in which sequencing was conducted using the SOLiD chemistry, and (**B**) without further filtering or using the Rare and True Filter in five families in which sequencing was performed using the TruSeq chemistry (see Results and discussion). Error bars indicate 95% confidence intervals. Data were processed according to *Pre-FAVR bioinformatic processing* then further FAVR filtering was applied as described in *FAVR bioinformatic processing* (see Methods).

Applied to SOLiD-derived data, the Rare and True Filter tool or both PE Bias Detector and Rare and True Filter tools resulted in mean O/Es (across families) of 1.00 and 1.05, respectively, compared with 1.95 both following no FAVR filtering and application of just the PE Bias Detector tool (95% CI= [0.80-1.20], [0.83-1.27], [1.75-2.14] and [1.77-2.14], respectively) (Figure 3A). For TruSeq-derived data, the O/E (across families) was 2.99 without FAVR filtering and 1.25 after Rare and True Filter (95% CI=[2.80-3.18] and [1.11-1.39], respectively) (Figure 3B).

The observations of O/Es close to 1 following application of FAVR methods provide confidence that the majority of remaining variants are likely to be genuine rare variants and not the result of sequencing or mapping artefacts. Findings using GATK v1.0.5336 showed very similar results (Additional file 2: Figure S2 A and C).

The encouraging results from our analyses using first cousin datasets were further substantiated by the following. Rare SNVs not reported in dbSNP131 and ‘called’ following FAVR processing of whole-exome data proved to be genuine according to Sanger sequence validation in 94% of cases (45/48). We assessed sensitivity of FAVR compared with pre-FAVR bioinformatic processing as outlined in Methods (note our assumption regarding the definition of genuine variants for the purposes of this sub-analysis and, to re-iterate, the difficulties faced with assigning appropriate reference data in the context of rare variant analyses). Our results projected that 100% (100/100) of rare, genuine variants ‘called’ would be maintained following FAVR processing.

The application of FAVR methods in the context of different sequencing chemistries and different mapping software indicate that similar benefits can be achieved across a range of analysis pipelines preceding the FAVR processing steps. The software that supports the FAVR methods outlined here is designed to be compatible with common sequence alignment [7] and variant caller file formats [8] that have been designed to standardise bioinformatic processing pipelines (Figure 1).

Application of Rare and True Filter and PE Bias Detector reduces the number of variants of possible interest per exome, in automated fashion and without compromising ‘true variant’ sensitivity. Further, Family Annotate Tool offers improvements over conventional methods to facilitate the rationalised optional shortlisting of variants based on assumptions relating to the nature of genetic variants and projected patterns of inheritance, respectively. The Rare and True Filter could be more broadly used in the study of any genetic-phenotypic analysis in which rare genetic variants are expected to contribute significantly to a given phenotype, by increasing their detection specificity.

When dbSNP135 (incorporating substantially more unvalidated MPS-generated variants) was used to filter variants instead of dbSNP131 for the above SOLiD analyses, we observed mean proportions of variants remaining of 0.67, 0.22 and 0.16 following application of PE Bias Detector, Rare and True Filter or both, respectively (95% CI=[0.56-0.79], [0.16-0.27] and [0.08-0.24]) (Additional file 2: Figure S2B). The mean O/Es of shared variants between first cousins (average across families) were 0.81 and 0.84 after applying the Rare and True Filter tool or both PE Bias Detector and Rare and True Filter, respectively, compared with 2.04 and 2.10 following no further filtering or application of just PE Bias Detector (95% CI=[0.64-0.99], [0.62-1.06], [1.87-2.22] and [1.94-2.25] respectively) (Additional file 2: Figure S2D). The significance of these findings is unclear since we cannot resolve what proportion of signal removed by filtering dbSNP135 represents real rare variants and what proportion represents artefacts.

**Figure 4:**
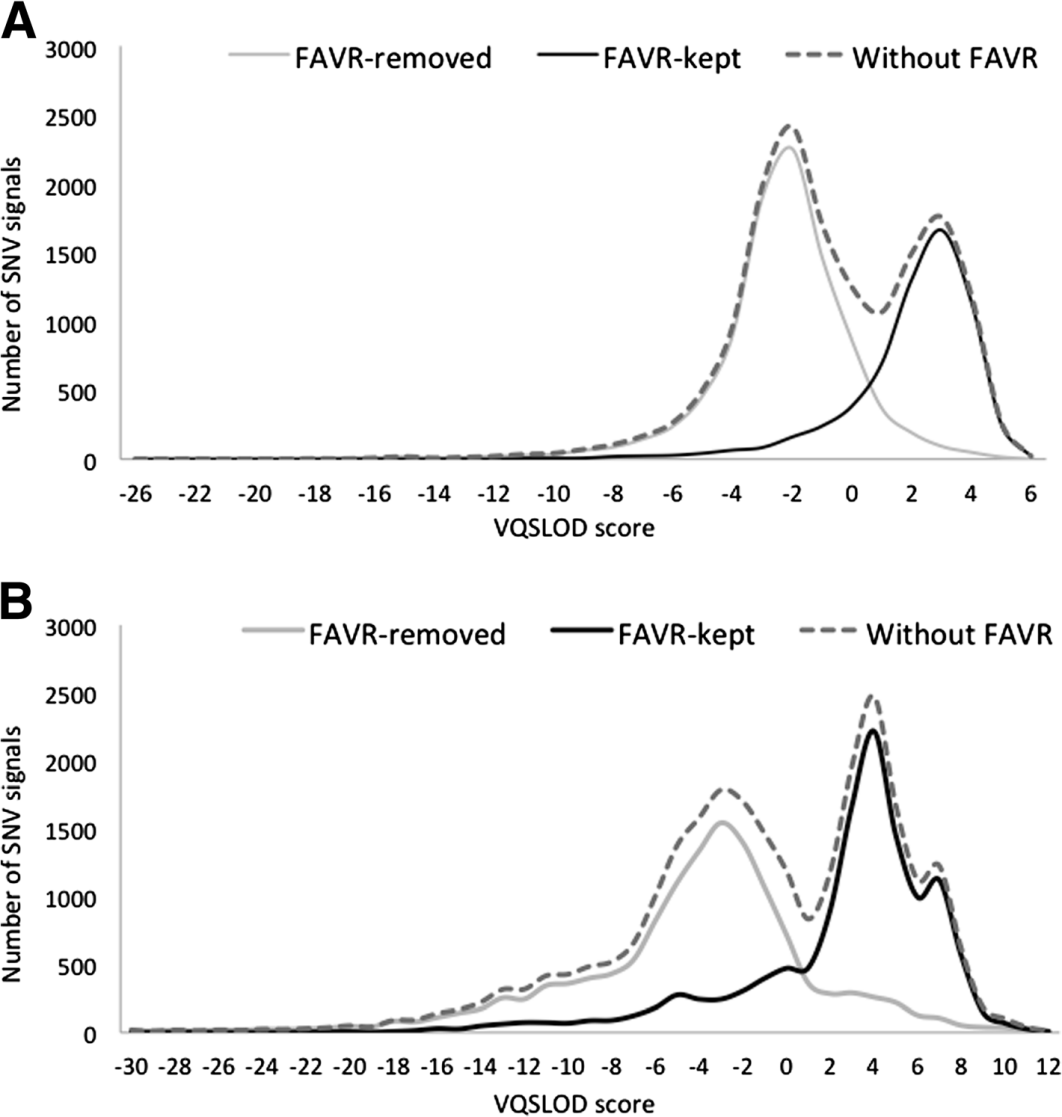
**Distribution of VQSLOD scores derived from the Variant Quality Score Recalibrator (GATK).** VQSLOD scores were obtained without FAVR processing and for FAVR-kept and FAVR-removed SNV signals after processing of SOLiD sequencing data (**A**) and TruSeq sequencing data (**B**), according to METHODS.

Figure 4 demonstrates the distribution of VQSLOD scores generated by the Variant Quality Score Recalibrator (GATK) for FAVR-kept and FAVR-removed SNV signals. The limited overlaps indicate reasonably good correlation between the confidence attributed to a variant called by GATK and the likelihood that it could be retained following FAVR processing. These data do suggest however that a VQSLOD score-based threshold to remove a high percentage of artefactual signals could result in a substantial proportion of true rare variants being unintentionally removed. Perhaps the best future strategy for rare SNV analyses may be to use similar principles to those embodied in machine-learned probabilistic approaches such as GATK in combination with FAVR methods. Under this type of strategy the VQSLOD score threshold may be relatively relaxed to achieve optimal balance of sensitivity and specificity. Of course, this could be tailored to suit requirements on an application-by-application basis.

These methods are not without limitations. As stated previously, FAVR methods do not currently separate apparently common variants into ‘true common’ or ‘artefact’ classifications, which restricts their utility to rare variant models unless used in conjunction with alternative approaches to study common genetic variation. The methods have also only been tested using the GATK variant caller and performance may vary when used with other variant callers. While we have used different mapping tools (Bioscope and Novoalign) in the context of different sequencing chemistries (SOLiD and Truseq), different mapping tools applied in conjunction with different variant calling software could also influence performance. To date, we have not included automated indel calling as part of FAVR methods, but intend to shortly and expect this to provide similar benefits to those observed for SNVs. FAVR software currently uses empirically-determined filtering thresholds that do not vary according to coverage depth at given positions or sequencing errors in particular contexts, for instance. In future iterations, we intend to incorporate such considerations to allow the FAVR principles to be applied in a more Bayesian approach.

It is possible that some of the principles outlined here may be extendable to the study of common variants. If we were to include thresholds governing the permissible range of variant read frequencies across a panel of specimens, for example, with measures of ‘non-called’ variant signals across the panel, we may be able to markedly increase non-rare variant calling specificity while only tolerably decreasing sensitivity. If so, such an approach could be useful in studies involving genomes that are not currently well-defined at population levels.

## Conclusions

The suite of tools presented here address the challenge of analysing the large amounts of data generated by MPS technologies, with emphasis on the study of rare genetic variants, in an accurate, efficient and time-effective manner. Handling and customisation of the whole FAVR suite does not require advanced computational skills or large computational resources and can be performed as part of routine data cleaning and interpretation pipelines. The methods can be applied across MPS sequencing platforms, accept commonly-used file formats, complement other commonly-used bioinformatic pipeline tools, and will be broadly useful for the study of rare genetic variants and their influence on phenotypes.

## Competing interest

The authors declare that they have no competing interests.

## Authors’ contributions

BJP designed and coded the software. TN-D and FO contributed to the methods design and conducted the testing. RB, SVT, KT, FH, DEG, AL and MCS contributed to data for testing. DJP conceived the filtering and annotation concepts. All authors contributed to the drafting of the manuscript. All authors read and approved the final manuscript.

## Supplementary Material

Additional file 1: Figure S1 Example of a typical artefact signal. Six individual alignment files are displayed. The variant has been ‘called’ only for the first individual whereas the variant signal also appears in other individuals. This assumed artefact signal was observed in systematic fashion across our dataset at a frequency higher than would be expected to be caused by sequencing chemistry errors. Click here for file

Additional file 2: Figure S2 Filtering of SOLiD sequencing data using dbSNP131 and dbSNP135. Mean (of first cousins) number of rare SNVs remaining without any further filtering and using the PE Bias Detector Tool only, the Rare and True Filter only, or both tools in five families, after filtering out common variants appearing in dbSNP131 (A) or dbSNP135 (B). Mean (across families) O/E number of shared SNVs, assuming first-cousins share 12.5% of their DNA (on average) without further filtering and using the PE Bias Detector Tool only, the Rare and True Filter only, or both tools in five families, after filtering on dbSNP131 (C) or dbSNP135 (D). Error bars indicate 95% confidence intervals (see Results and discussion). Data were processed according to *Pre-FAVR bioinformatic processing* and further FAVR filtering was applied as described in *FAVR bioinformatic processing* (see Methods). Click here for file

Additional file 3: Table S1 Total number of variants found in each individual and observed number of shared variants between first cousins pairs, at the different stages of filtering. N/A indicates non-applicable. Data were processed according to *Pre-FAVR bioinformatic processing* and further FAVR filtering was applied as described in *FAVR bioinformatic processing* (see Methods). Click here for file
