# Supplementary figures and images for: FAVR (Filtering and Annotation of Variants that are Rare): methods to facilitate the analysis of rare germline genetic variants from massively parallel sequencing datasets
Source: BMC Bioinformatics. 2013 Feb 25;14:65. doi: 10.1186/1471-2105-14-65 (PMC3599469; doi:10.1186/1471-2105-14-65)

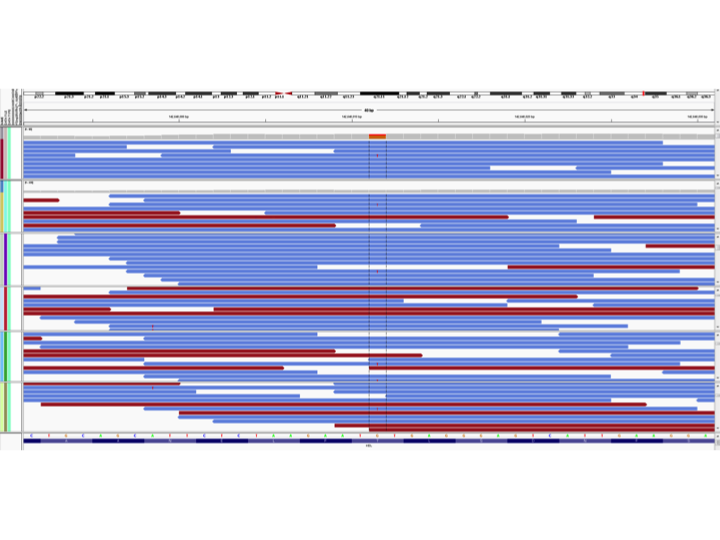

Supplement: Additional file 1: Figure S1 — Example of a typical artefact signal. Six individual alignment files are displayed. The variant has been ‘called’ only for the first individual whereas the variant signal also appears in other individuals. This assumed artefact signal was observed in systematic fashion across our dataset at a frequency higher than would be expected to be caused by sequencing chemistry errors. [file 1471-2105-14-65-S1.tiff]

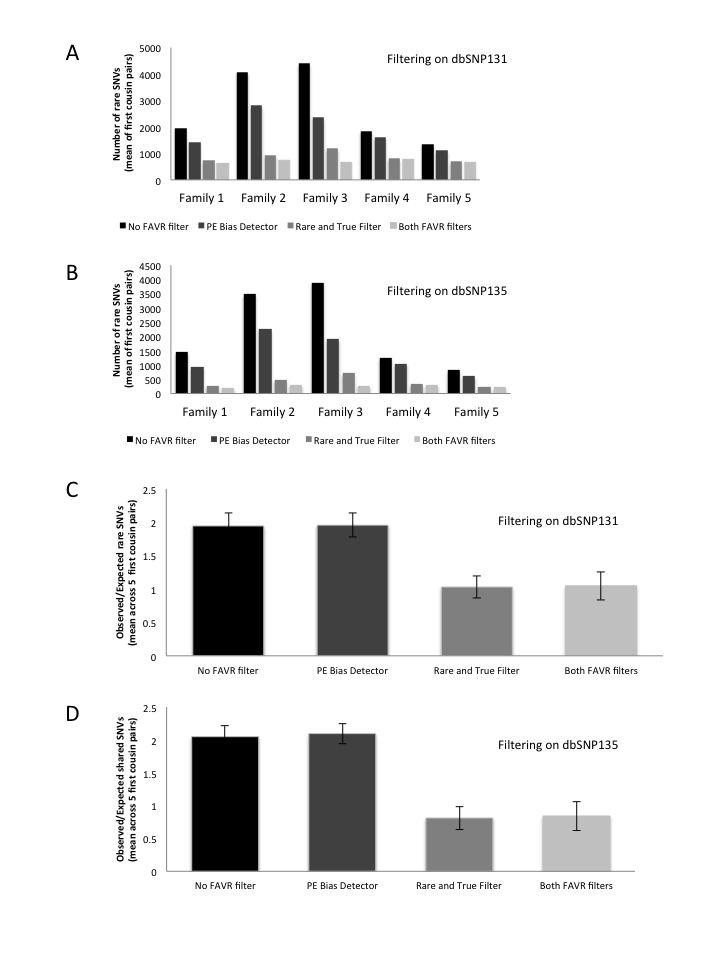

Supplement: Additional file 2: Figure S2 — Filtering of SOLiD sequencing data using dbSNP131 and dbSNP135. Mean (of first cousins) number of rare SNVs remaining without any further filtering and using the PE Bias Detector Tool only, the Rare and True Filter only, or both tools in five families, after filtering out common variants appearing in dbSNP131 (A) or dbSNP135 (B). Mean (across families) O/E number of shared SNVs, assuming first-cousins share 12.5% of their DNA (on average) without further filtering and using the PE Bias Detector Tool only, the Rare and True Filter only, or both tools in five families, after filtering on dbSNP131 (C) or dbSNP135 (D). Error bars indicate 95% confidence intervals (see Results and discussion). Data were processed according to Pre-FAVR bioinformatic processing and further FAVR filtering was applied as described in FAVR bioinformatic processing (see Methods). [file 1471-2105-14-65-S2.tiff]
