# Supplementary material for: FAVR (Filtering and Annotation of Variants that are Rare): methods to facilitate the analysis of rare germline genetic variants from massively parallel sequencing datasets
Source: BMC Bioinformatics. 2013 Feb 25;14:65. doi: 10.1186/1471-2105-14-65 (PMC3599469; doi:10.1186/1471-2105-14-65)
Supplement: Additional file 3: Table S1 — Total number of variants found in each individual and observed number of shared variants between first cousins pairs, at the different stages of filtering. N/A indicates non-applicable. Data were processed according to Pre-FAVR bioinformatic processing and further FAVR filtering was applied as described in FAVR bioinformatic processing (see Methods). [file 1471-2105-14-65-S3.pdf]

		No FAVR filtering	Observed shared	PE Bias Detector	Observed shared	Rare and True Filter	Observed shared	Both FAVR filters	Observed shared
Family 1	Relative 1	1840	429	1457	319	739	88	684	85
	Relative 2	2020		1372		698		596	
Family 2	Relative 1	3198	1097	2329	763	821	119	689	99
	Relative 2	4680		3109		995		773	
Family 3	Relative 1	4407	1078	2497	561	1198	86	796	52
	Relative 2	4199		2177		993		540	
Family 4	Relative 1	1861	456	1623	407	810	120	769	119
	Relative 2	1692		1518		803		772	
Family 5	Relative 1	1378	278	1204	237	751	101	720	101
	Relative 2	1283		1006		642		595	
Family 6	Relative 1	2490	1074	N/A	N/A	1094	218	N/A	N/A
	Relative 2	2572				1173			
Family 7	Relative 1	2549	919	N/A	N/A	1111	150	N/A	N/A
	Relative 2	2326				1073			
Family 8	Relative 1	2153	778	N/A	N/A	1132	186	N/A	N/A
	Relative 2	2223				1262			
Family 9	Relative 1	2297	836	N/A	N/A	1072	168	N/A	N/A
	Relative 2	2308				1039			
Family 10	Relative 1	2474	871	N/A	N/A	1357	189	N/A	N/A
	Relative 2	2495				1368			
